# Association of Molecular Detections of Microsporidia in Stool Samples with Clinical and Immunological Parameters in Ghanaian HIV Patients

**DOI:** 10.3390/pathogens13121053

**Published:** 2024-11-29

**Authors:** Hagen Frickmann, Fred Stephen Sarfo, Betty Roberta Norman, Albert Dompreh, Shadrack Osei Asibey, Richard Boateng, Edmund Osei Kuffour, Konstantin Tanida, Veronica Di Cristanziano, Torsten Feldt, Kirsten Alexandra Eberhardt

**Affiliations:** 1 Department of Microbiology and Hospital Hygiene, Bundeswehr Hospital Hamburg, 22049 Hamburg, Germany; 2 Institute for Medical Microbiology, Virology and Hygiene, University Medicine Rostock, 18057 Rostock, Germany; 3 Department of Medicine, Komfo Anokye Teaching Hospital, Kumasi 00233, Ghana; stephensarfo78@gmail.com (F.S.S.); branorman@yahoo.com (B.R.N.); shakosbey19@gmail.com (S.O.A.); 4 Kwame Nkrumah University of Science and Technology, Kumasi 00233, Ghana; 5 Department of Clinical Microbiology, Komfo Anokye Teaching Hospital, Kumasi 00233, Ghana; adompreh@gmail.com (A.D.); richardboateng166@gmail.com (R.B.); 6 Laboratory of Retrovirology, The Rockefeller University, New York, NY 10065, USA; eosei@rockefeller.edu; 7 Institute of Medical Microbiology, Virology and Hygiene, University Medical Center Hamburg-Eppendorf (UKE), 20251 Hamburg, Germany; k.tanida@uke.de; 8 Institute of Virology, Faculty of Medicine and University Hospital Cologne, University of Cologne, 50937 Cologne, Germany; veronica.di-cristanziano@uk-koeln.de; 9 Clinic of Gastroenterology, Hepatology and Infectious Diseases, University Hospital Düsseldorf, 40225 Düsseldorf, Germany; torsten.feldt@med.uni-duesseldorf.de; 10 Department of Tropical Medicine, Bernhard Nocht Institute for Tropical Medicine & I. Department of Medicine, University Medical Center Hamburg-Eppendorf, 20359 Hamburg, Germany; k.eberhardt@bnitm.de

**Keywords:** microsporidia, *Encephalitozoon*, *Enterocytozoon*, HIV, immunosuppression, Western Africa, interaction

## Abstract

Although the etiological relevance of the detection of microsporidia in human stool samples remains uncertain, the immunological status of patients has been posited as an important determinant of potential clinical impact of these parasites. To further assess the interplay between the epidemiology of microsporidia and immunological markers, we conducted a study utilizing real-time PCR targeting *Enterocytozoon bieneusi*, *Encephalitozoon cuniculi*, *Encephalitozoon hellem*, and *Encephalitozoon intestinalis*, combined in a single fluorescence channel. The study involved a cohort of 595 clinically and immunologically well-characterized Ghanaian HIV patients, alongside 82 HIV-negative control individuals from Ghana. While microsporidial DNA was absent in HIV-negative controls, among people living with HIV, its prevalence was inversely correlated with CD4+ lymphocyte counts: 6.0% in those with >500 cells/µL, 9.5% in those with 200–499 cells/µL, 13.8% in those with 50–199 cells/µL, and 27.5% in those with <50 cells/µL, respectively. Correspondingly, microsporidia were more frequently detected in HIV patients who were not receiving antiretroviral therapy. There were no associations with clinical symptoms including gastroenteritis with the exception of a non-significant trend towards weight loss. HLA-DR+CD38+ on CD4+ T lymphocytes, a marker of immune activation, as well as Ki67, a marker of cell proliferation, were increased on CD4+ T lymphocytes in HIV patients with microsporidia, suggesting an immune response may be triggered. In conclusion, our assessment indicates a higher prevalence of microsporidia in the stool of Ghanaian HIV patients, which varies with their immunological status. However, given the lack of clear associations with clinical symptoms, the detection of microsporidia in the stool of HIV patients needs to be cautiously interpreted in clinical settings.

## 1. Introduction

Infections with human immunodeficiency virus (HIV) predispose individuals to infections with agents of low-virulence, opportunistic microorganisms like microsporidia. Microsporidia are protozoon-like fungi that are commonly associated with HIV-induced immunosuppression. Of note, the term “microsporidia” is a non-taxonomic description of intracellular microorganisms of the phylum Microspora [1,2]. Globally, a pooled prevalence of 11.5% microsporidia has been estimated for people living with HIV (PLWH), with higher rates observed in resource-poor settings, particularly sub-Saharan Africa [3,4,5]. The first case of a human microsporidial infection was reported in 1985 [6]. Diarrhea is frequently observed in patients positive for both HIV and microsporidia [3], although symptomatic disease rates have considerably dropped due to increased availability of anti-retroviral therapy [7]. Next to microsporidia-induced diarrhea, systemic dissemination with non-enteric lesions like keratitis, keratoconjunctivitis, sinusitis, tracheobronchitis with associated shortness of breath, cerebritis and encephalitis, interstitial nephritis, hepatitis, cholecystitis, peritonitis osteomyelitis, myositis and even fatal courses have been described [6,7,8,9,10,11]. Chronic diarrhea and even wasting, in particular, have been associated with *Enterocytozoon bieneusi* [9,10] and less frequently with *Encephalitozoon* (formerly *Septata*) *intestinalis*, which can also show systemic dissemination [9]. A recent meta-analysis [12] reported that the ratio of *E. bieneusi* carriers with and without diarrhea was 3:1, and highlighted risk factors such as male sex, being a teenager, and living in rural areas or coastal climates. Other types of immunosuppression, such as chemotherapy- or transplantation-induced variants, as well as very low or high age or medical conditions like diabetes and malignant disease, have been associated with microsporidiasis [7,8]. Additionally, microsporidia may cause self-limiting traveler’s diarrhea, and even immunocompetent individuals can intermittently shed microsporidia in feces and urine without symptoms [7,13]. In cases of severe immunosuppression, debilitating symptoms such as weight loss, abdominal pain, anorexia, nausea, and diarrhea, and high mortality risks have been observed [8,14]. For individuals with immunosuppression following transplantation, screening for microsporidia is recommended if they have persistent diarrhea or chronic fever of unknown origin [15].

Ingestion occurs via the oral route [13]. Ingested spores can invade their host cells with the help of a highly specialized invasion apparatus called the polar tube [8]. The genome of microsporidia is described as compact and reduced [16] and some features of their ribosomes resemble even procaryotes [2]. Consequently, phylogenetic analyses assigned microsporidia to early branches in the evolutionary line of eukaryotes [2]. The microorganisms’ metabolism and reproduction largely depend on host substrates [17]. Excess death of enterocytes and inflammation due to cellular infection result in small intestinal injury, malabsorption, diarrhea, and weight loss [9]. Next to epithelial cells, mesenchymal and neural cells can be affected by microsporidial infection as well [18]. Low CD4+ T-lymphocyte counts have been associated with more severe disease manifestation, more atypical disease and a greater risk of disseminated infection [19]. Nevertheless, and as known from mouse experiments, CD8+ T-lymphocytes in gut-associated lymphoid tissue are key for protecting against infection [20].

Diagnostic approaches for the identification of microsporidial colonization or infection comprise microscopic stool examination with or without electron microscopy and immunofluorescence microscopy, molecular diagnostic techniques, and specific stains of small bowel biopsies [10,13], such as modified trichrome blue stain [14], optical brighteners [15], or immunofluorescence staining [21,22]. The risk of overlooking microsporidia results from their small size, intracellular persistence and poor stainability with various standard tissue staining protocols [15]. Next to tissue specimens, diagnostic assessment of other samples like stool, duodenal aspirates, urine, sputum, nasal discharge, bronchoalveolar lavage fluid or other secretions and conjunctival smears can be promising depending on the site of infection [1,9,10,16]. Of note, confusion of microsporidial cysts with yeasts in case of microsporidial myositis in severely immunocompromised patients has been described [23]. PCR-based identification of microsporidia has been introduced as early as in the 1990s [1,9,24]. Restriction fragment length polymorphism analysis has been used to differentiate between the various types of microsporidial microorganisms [16].

Specific therapy apart from the improvement of the host’s immunological condition is poorly standardized [14,17,18], potential beneficial effects of albendazole on the clinical course of *Enterocytozoon bieneusi* and *Encephalitozoon* spp. infections have been inconsistently reported [11,14,17]. While *Encephalitozoon intestinalis* has been shown to therapeutically respond well to albendazole [17] and some beneficial effects have also been suggested for human *E. cuniculi* infections [25], fumagillin derivates were associated with therapeutic effects against *E. bieneusi* [17]. Promising effects of polyamine inhibitors and thalidomide against microsporidia have also been demonstrated in vitro and partly in vivo [17]. Water safety is crucial for infection prevention and control [26].

Epidemiological information on microsporidial carriage in Ghanaian PLWH is scarce, although infections in mammals in Ghana were reported as early as the 1990s [27]. In a recent study by our group [28], microsporidial DNA was detected in 78 out of 903 (8.6%) assessed Ghanaian stool samples. Although this result comes close to the general estimation of microsporidial prevalence in PLWH in sub-Saharan Africa as stated above [3], those data have to be interpreted with care due to the inclusion of repeated sampling from identical patients. Hygiene challenges like contaminated drinking water have recently been suggested to contribute to the spread of microsporidia to Ghanaian individuals [29], matching previous experience from other regions [26].

To address the knowledge gap on HIV-associated microsporidiasis in Ghanaian individuals, a cross-sectional study with stool samples from Ghanaian PLWH was conducted using a real-time PCR assay covering the species *Enterocytozoon bieneusi*, *Encephalitozoon cuniculi*, *Encephalitozoon hellem*, and *Encephalitozoon intestinalis*. The findings were correlated with clinical disease and immunosuppression-associated parameters.

## 2. Materials and Methods

### 2.1. Study Cohort and Data Collection

As part of a study investigating associations of gastrointestinal and other pathogens with immunological parameters in HIV-positive and negative adults in Ghana, consecutive HIV positive patients presenting to the HIV outpatient department of the Komfo Anokye Teaching Hospital (Kumasi, Ghana) were offered participation [30]. An HIV-negative control group was recruited over the same time period of 12 months. All participants provided a written informed consent prior to enrolment. Demographic, socioeconomic, and clinical data were recorded using standardized questionnaires, which were completed by trained study personnel.

### 2.2. Laboratory Methods

Venous blood samples were collected, and the analysis of CD4+ T cell count was performed locally using a FACSCalibur flow cytometer (Becton Dickinson, Mountain View, CA, USA). HIV-1 viral load was measured using the Real-Time HIV-1 PCR system (Abbott Diagnostics, Wiesbaden, Germany).

Peripheral blood mononuclear cells (PBMCs) were isolated by centrifugation of heparinized venous blood on a Ficoll/Hypaque (Biocoll Seperating Solution, Biochrom AG, Berlin, Germany) density gradient. Cells were washed in phosphate-buffered saline and resuspended in Roswell Park Memorial Institute 1640 medium (both Gibco Invitrogen, Carlsbad, CA, USA) supplemented with heat-inactivated fetal calf serum (Biochrom AG, Berlin, Germany). PBMCs were cryopreserved and shipped to Germany on liquid nitrogen. Cell surface markers for immune activation were stained as described in a previous work [31]. Flow cytometric data were acquired using the LSRII flow cytometer (BD Biosciences, Heidelberg, Germany) and analyzed using FlowJo version 9.6.2 (formerly Tree Star, San Carlos, CA, USA, now Becton Dickinson, Franklin Lakes, NJ, USA).

Fresh native stool sample aliquots were deep frozen at −80 °C prior to nucleic acid purification. Nucleic acids extraction was conducted using the QIAamp stool DNA mini kit (Qiagen, Hilden, Germany) according to the manufacturer’s instructions and eluates were again stored at −80 °C prior to real-time PCR analysis. The applied real-time in-house PCR assay for microsporidia targeted a 394-base pair sequence of the small subunit ribosomal RNA (SSU rRNA) gene of the four species *Enterocytozoon bieneusi*, *Encephalitozoon cuniculi*, *Encephalitozoon hellem*, and *Encephalitozoon intestinalis* with reported relevance for human infections as described recently [28]. For this assay, sensitivity of 97.4% and specificity of 99.1% had been estimated with a limit of detection of <50 copies per µL eluate [28]. The in-house assay was conducted on magnetic induction cyclers (MIC, Bio Molecular Systems Ltd., London, UK) using 20 µL volumes including 2 µL eluate each. The applied oligonucleotides comprised the forward primers 1 and 2 (5′-CACCAGGTTGATTCTGCCTGA-3′, 5′-TCCGGAGAGGGAGCCTGAG-3′), the reverse primers 1-4 (5′-GCTTGCCCTCCAATTGCTTC-3′, 5′-GACTTGCCCTCCAATCACATG-3′, 5′-CCGACTTGCCCTCCAGTAAA-3′, 5′-CTTGGCCTCCAATCAATCTCG-3′) and the hybridization probe 5′-TGGCAGCAGGCGCGAAACTTGT-3′. The main component of the PCR reaction mix was the HotStarTaq Mastermix (Qiagen, Hilden, Germany) with a final Mg^2+^ concentration of 6 mM. The concentrations of the oligonucleotides used for the real-time PCR were 40 nM for each primer and 10 nM for the probe. A PCR grade water-based negative control and a positive control based on a plasmid containing the microsporidial sequence 5′-AACACGGACCCACCAGGTTGATTCTGCCTGACGTAGATGCTAGTCTCTGAGATTAAGCCATGCATGTCAGTGAAGCCTTACGGTGGAACGGCGAACGGCTCAGTAATGTTGCGGTAATTTGGTCTCTGTGTGTAAACTAACCACGGTAACCTGTGGCTAAAAGCGGAGAATAAGGCGCAACCCTATCAGCTTGTTGGTAGTGTAAAGGACTACCAAGGCCATGACGGGTAACGGGAAATCAGGGTTTGATTCCGGAGAGGGAGCCTGAGAGATGGCTCCCACGTCCAAGGACGGCAGCAGGCGCGAAACTTGTCCACTCCTTACGGGGGAGACAGTCATGAGACGTGAGTATAAGACCTGAGTGTAAAGACCTTAGGGTGAAGCAATTGGAGGGCAAGCTTTGGTGCCAGCAGC-3′ (NCBI GenBank accession number AF023245) inserted in a pEX-A128 vector backbone were included in each run. Sample inhibition was controlled with the help of a Phocid herpes virus DNA-specific real-time PCR as detailed elsewhere [32]. The real-time PCR run profile consisted of initial denaturation at 95 °C for 15 min followed by 50 cycles of denaturation at 95 °C for 15 s, annealing at 60 °C for 30 s and amplification for 30 s at 72 °C. Final, cooling down to 40 °C for 20 s was conducted.

### 2.3. Statistical Analysis

Statistical analyses were performed using R (version 4.3.0, R Foundation for Statistical Computing, Vienna, Austria). Categorical variables were compared using either the χ2 test or the Fisher exact test, as appropriate. Continuous variables were expressed as median (interquartile range, IQR) or mean ± standard deviation (SD) and compared using the Wilcoxon rank sum test or the unpaired Student’s *t*-test. The Spearman rank correlation coefficient ρ was calculated to determine the relation existing between continuous variables. Two-sided *p*-values were presented, and statistical significance was determined at α = 5%.

## 3. Results

### 3.1. Prevalence of Microsporidia in Fecal Specimens in the Study Population

**Figure 1:**
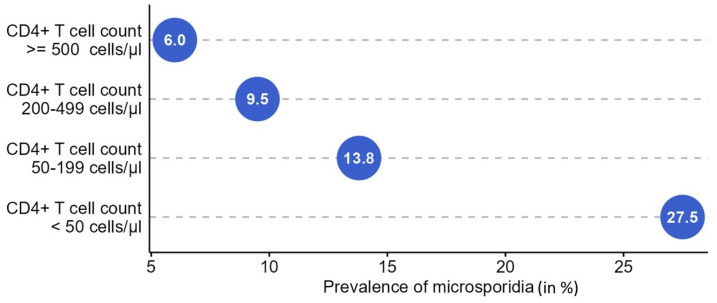
Prevalence of microsporidia (*x*-axis in %) in HIV positive participants according to the CD4+ T cell count.

A total of 1095 HIV-positive and 107 HIV-negative individuals were included in this study. Residual stool samples for microsporidia testing were available for 595 HIV positive and 82 HIV negative participants. While microsporidia were not detected in stool samples of any of the HIV negative volunteers, the overall prevalence was 10.6% (*n* = 63/595) in the group of PLWH. The Cleveland’s plot demonstrates an inverse relationship between microsporidial prevalence and CD4+ T lymphocyte count in HIV positive participants: In subjects with CD4+ T cell counts above 500 cells/µL, the prevalence was 6.0% (n = 11/182), it was 9.5% (n = 21/221) in individuals with counts between 200 and 499 cells/µL, 13.8% (n = 17/123) in HIV positives with CD4+ T cells between 50 and 199 cells/µL and 27.5% (n = 14/51) in PLWH with CD4+ T cell counts below 50 cells/µL (*p* < 0.001, Figure 1).

### 3.2. Comparison of Demographic, Socioeconomic and Clinical Characteristics of the HIV Cohort According to Microsporidia Status

HIV-positive participants with or without detected microsporidia in fecal specimens were not different regarding age or sex (Table 1). In addition, no significant differences in socioeconomic parameters were found between both groups. HIV-positive participants co-infected with this pathogen were less often exposed to cART (23.8% vs. 43.8%, *p* = 0.004). If exposed to cART, patients with microsporidia were treated for a longer time period than those without this co-infection (84.5 [39.8–96.4 IQR] vs. 50.9 [23.1–77.3 IQR] months, *p* = 0.040). Gastrointestinal complaints or other clinical symptoms were not reported to show different proportions in comparison of PLWH with and without microsporidia, with the exemption of a non-significant tendency for weight loss (34.9% vs. 23.0%, *p* = 0.053) in case of colonization with microsporidia.

### 3.3. Comparison of Virological and Immunological Characteristics of the HIV Cohort According to Microsporidia Status

Among participants who were on cART, 6.2% (n = 15/242) were found to be colonized with microsporidia. However, there were no significant differences in virological or immunological parameters between those with microsporidia and those without.

On the other hand, a proportion of 14.2% (n = 48/339, *p* = 0.004) of cART naïve patients were carrying microsporidia. Co-infected patients in this subgroup had a significantly higher HIV-1 median viral load in log10 copies/mL (5.5 [5.2–5.9 IQR] vs. 5.0 [4.2–5.6 IQR], *p* < 0.001, Table 2) and a correspondingly lower CD4+ T cell count/µL (102 [415–366 IQR] vs. 246 [104–464 IQR], *p* = 0.005). The CD4+/CD8+ T cell ratio, which is inversely associated with immune activation in HIV, was significantly lower in microsporidia carriers (0.1 [0.1–0.3 IQR] vs. 0.3 [0.1–0.5 IQR], *p* = 0.009). HIV positive individuals with microsporidia carriage had a significantly higher expression of HLA-DR+CD38+ on CD4+ T lymphocytes as additional markers of immune activation (36.1 [26.8–45.8 IQR] vs. 23.4 [13.7–35.3 IQR], *p* = 0.012). Furthermore, the marker of cell proliferation Ki67+ on CD4+ T lymphocytes was elevated in individuals with detected microsporidia compared to those without those pathogens (55.0 [45.5–63.1 IQR] vs. 15.8 [9.7–40.7], *p* = 0.028). No differences were found in the expression of markers of immune exhaustion and terminal differentiation or of markers of immune activation on CD8+ T lymphocytes when comparing cART naïve HIV patients with and without microsporidia.

### 3.4. Correlations of Cyle Threshold (Ct) Values with CD4+ T Cell Count, CD4+/CD8+ T Cell Ratio, and HIV Viral Load

The correlation analysis of microsporidia-specific cycle threshold (Ct) values in real-time PCR and HIV-1 viral load, CD4+ T cell count as well as CD4+/CD8+ T cell ratio in microsporidia-positive participants revealed a significant correlation for CD4+ lymphocyte count and the CD4+/CD8+ T cell ratio and the Ct value of microsporidia and an inverse correlation for the HIV-1 viral load and the Ct values of microsporidia (ρ = 0.29, *p* = 0.023, ρ = 0.35, *p* = 0.009, and ρ = −3.50, *p* = 0.007, respectively).

## 4. Discussion

The study was conducted to assess epidemiological and immunological features of Ghanaian PLWH carrying microsporidia in their intestinal tract, and it yielded several key results.

First, the abundance of detected microsporidial DNA in Ghanaian stool samples was negatively correlated with the recorded CD4+ T-lymphocyte counts. This is well in line with previous reports [19], stressing the importance of immunosuppression for the spread and disease severity of microsporidial infections in human individuals. Of note, microsporidial DNA was not detected in any of the stool samples of non-HIV-infected Ghanaians, although the respective number of assessments was quite low, limiting the interpretability of this result.

While hygiene-related and socioeconomic variables played a role for the prevalence of microsporidia in previous assessments [3], this was not the case for the presented Ghanaian cohort. In particular, a preference for young age and male sex as reported elsewhere for microsporidial infections could not be confirmed in our study [12].

In concordance with extant literature, we observed associations between patients’ immunological state, use of anti-retroviral therapy and molecular detection of microsporidia. More robust immunologic status and use of antiretroviral therapy were protective against microsporidial colonization or infection [7]. It is noteworthy, however, that microsporidial detection rates were again on the rise in individuals on long-term antiretroviral therapy. It can only be speculated that selection of retroviral resistance, which is known to be common in Ghana [33,34,35,36,37,38,39,40,41,42], with potentially deleterious effects on the patients’ immunological state might be more pronounced in PLWH on long-term treatment.

Interestingly, clinical symptoms of PLWH with or without microsporidial detections in their stool samples were virtually indistinguishable. A non-significant tendency for more weight loss in PLWH with microsporidia in stool might simply reflect the association of microsporidial prevalence and severity of immunosuppression. This finding impressively confirms previous demonstrations of only a minority of enteric microsporidial infections being associated with infectious gastroenteritis [12]. Further and as detailed elsewhere [43,44], generally high prevalence of gastroenteric pathogens in Ghanaian individuals necessarily interferes with potential effects of the detected microsporidia on the patients’ health and wellbeing.

Focusing on the immunological state of PLWH with microsporidia, they had higher viral loads, lower CD4+ T-lymphocyte counts and reduced CD4+/CD8+ T-lymphocyte ratios. The finding is well in line with previous experience [19]. Further, markers of immune-activation like HLA-DR+CD38+ and of cellular proliferation like Ki67+ on CD4+ T-lymphocytes were increased in case of microsporidial detections, while specific effects on determinants of immune exhaustion could not be demonstrated. Also interesting, effects on CD8+ T-lymphocyte activation were not seen, although these cells are considered as a major line of defense against microsporidial infections [20]. This finding might suggest that a considerable share of the microsporidial detections just indicated colonization rather than infections.

Finally, semiquantitative analysis based on cycle threshold values correlated high HIV loads with high microsporidial quantities in stool, while robust immunological conditions as indicated by high CD4+ T-lymphocyte counts and high CD4+/CD8+ T-lymphocyte rates were associated with low microsporidial loads. Assuming that low microsporidial loads in patient stool might indicate microsporidial colonization rather than infection, this finding again confirms considerable effects of the patients’ immunological state on the dissemination or immunological control of microsporidia [19,20].

The study has a number of limitations. First of all, the applied real-time PCR assay covered only a defined number of microsporidial species and did not discriminate between them. Second, the cohort’s sample size was limited, making the interpretation of analyses based on sub-stratification with partially very small sub-cohorts challenging. Third, potentially abundant additional enteric co-infections [43,44] might have interfered with the effects of the detected microsporidia. Fourth, due to a low variability of applied anti-retroviral therapy (ART) schemes with 96.7% of patients on ART taking nucleoside reverse transcriptase inhibitor (NRTI)/non-nucleoside reverse transcriptase inhibitor (NNRTI) combinations and the remaining 3.3% taking NRTI/protease inhibitor (PI) combinations [30], stratification on ART medication level was not performed. Similarly, there were no records of co-medication in a frequency justifying a statistical analysis. Fifth, socioeconomic parameters were indirectly assessed and stratified, with access to technical equipment like televisions and cars used as surrogate parameters for the patients’ economic potential. Other parameters like level of education, presence of animals like, e.g., domestic birds nearby, quality of the household and socioeconomical quartile might have been more informative and should be considered for future assessments.

## 5. Conclusions

Despite the limitations, this study provides valuable insights into the epidemiology and immunology of microsporidial enteric colonization or infections in Ghanaian PLWH. The assessment confirmed more prominent effects of the patients’ immunological state on the likelihood of the detection of microsporidia compared to socioeconomic factors. Altogether, the effect of microsporidial enteric detections on the patients’ clinical presentation seemed negligible in the assessed Ghanaian cohort.

## Figures and Tables

**Table 1:** Demographics, socioeconomic parameters, medical treatment and clinical symptoms in HIV infected individuals according to microsporidia status.

	Variable	HIV Positive Microsporidia Positive, n = 63 (10.6%)	HIV Positive Microsporidia Negative, n = 532 (89.4%)	*p*-Value
Demographics	Age in years ± SD	39.7 ± 8.3	40.6 ± 9.7	0.450
	Female, n (%)	43 (68.3)	391 (75.5)	0.275
Socioeconomic parameters	Access to tap water, n (%)	30 (47.6)	280 (54.1)	0.405
	Electricity in household, n (%)	59 (93.7)	483 (93.2)	1.000
	Television in household, n (%)	50 (79. 4)	428 (82.6)	0.642
	Refrigerator in household, n (%)	43 (68.3)	380 (73.4)	0.478
	Owning a car, n (%)	9 (14.3)	49 (9.5)	0.325
Medical therapy	Intake of cART, n (%)	15 (23.8)	227 (43.8)	0.004
	Months since initiation of cART, median (IQR)	84.5 (39.8–96.4)	50.9 (23.1–77.3)	0.040
Clinical symptoms during the last six months	Gastrointestinal symptoms, n (%)	11 (17.5)	71 (13.7)	0.538
	Fever, n (%)	5 (12.8)	34 (9.5)	0.705
	Cough, n (%)	9 (14.3)	55 (10.6)	0.506
	Weight loss, n (%)	22 (34.9)	119 (23.0)	0.053

SD—standard deviation; cART—combined antiretroviral therapy; IQR—Interquartile range.

**Table 2:** Virological and immunological parameters according to microsporidia and cART status.

Variable	HIV Positive cART Exposed			HIV Positive cART Naïve		
	Microsporidia Positive, Median (IQR), n = 15 (6.2%)	Microsporidia Negative, Median (IQR), n = 227 (93.8%)	*p*-Value	Microsporidia Positive, Median (IQR), n = 48 (14.2%)	Microsporidia Negative, Median (IQR), n = 291 (85.8)	*p*-Value
Viral load, log10 copies/ml	1.6 (0.0–4.0)	1.6 (0.0–1.8)	0.155	5.5 (5.2–5.9)	5.0 (4.2–5.6)	<0.001
CD4+ T cell count/µL	414.0 (298.0–592.5)	485.0 (308.2–699.8)	0.397	102.0 (41.0–365.8)	246.0 (104.0–464.0)	0.005
CD8+ T cell count/µL	902.5 (610.8–1108.5)	930.0 (655.0–1331.2)	0.500	1092.0 (566.0–1606.0)	992.0 (631.0–1495.0)	0.933
CD4+/CD8+ T cell ratio	0.5 (0.4–1.1)	0.5 (0.4–0.8)	0.507	0.1 (0.1–0.3)	0.3 (0.1–0.5)	0.009
HLA-DR+ CD38+ CD4+ (%)	10.2 (8.7–20.2)	12.0 (6.6–19.3)	0.829	36.1 (26.8–45.8)	23.4 (13.7–35.3)	0.012
HLA-DR+ CD38+ CD8+ (%)	28.6 (18.2–38.2)	28.0 (20.8–43.3)	0.697	49.0 (41.2–66.6)	51.8 (42.8–64.7)	0.829
CD57+ CD4+ (%)	16.2 (11.2–21.9)	14.2 (8.3–20.7)	0.642	16.4 (8.7–24.5)	14.8 (8.8–26.5)	0.762
CD57+ CD8+ (%)	47.9 (36.4–58.2)	51.2 (42.1–59.2)	0.506	49.4 (42.4–55.7)	44.4 (34.7–55.6)	0.169
PD-1+ CD4+ (%)	31.9 (20.0–42.5	30.6 (19.6–41.9)	0.623	43.8 (32.1–54.8)	36.4 (24.7–56.8)	0.132
PD-1+ CD8+ (%)	32.1 (18.5–35.6)	25.9 (15.7–39.0)	0.585	41.9 (28.1–45.0)	39.5 (26.5–50.7)	0.772
Ki67+ CD4+ (%)	12.1 (8.7–13.5)	7.1 (5.1–16.4)	0.488	55.0 (45.5–63.1)	15.8 (9.7–40.7)	0.028
Ki67+ CD8+ (%)	9.9 (9.8–11.7)	6.7 (3.1–18.7)	0.396	29.6 (15.4–43.9)	11.9 (8.1–18.1)	0.051

cART—combined antiretroviral therapy; IQR—Interquartile range.

## Data Availability

All relevant data are provided in the manuscript. Raw data can be made available upon reasonable request.
